# Association between *PPAR-γ2* Pro12Ala genotype and insulin resistance is modified by circulating lipids in Mexican children

**DOI:** 10.1038/srep24472

**Published:** 2016-04-14

**Authors:** Carolina Stryjecki, Jesus Peralta-Romero, Akram Alyass, Roberto Karam-Araujo, Fernando Suarez, Jaime Gomez-Zamudio, Ana Burguete-Garcia, Miguel Cruz, David Meyre

**Affiliations:** 1 Department of Clinical Epidemiology and Biostatistics, McMaster University, Hamilton, ON, Canada; 2 Medical Research Unit in Biochemistry, Hospital de Especialidades, Centro Médico Nacional Siglo XXI del Instituto Mexicano del Seguro Social, Mexico City, Mexico; 3 Health Promotion Division, Instituto Mexicano del Seguro Social, Mexico City, Mexico; 4 Centro de investigación sobre enfermedades infecciosas. Instituto Nacional de Salud Pública. Cuernavaca, Morelos, Mexico; 5 Department of Pathology and Molecular Medicine, McMaster University, Hamilton, ON, Canada

## Abstract

The Pro12Ala (rs1801282) polymorphism in peroxisome proliferator-activated receptor-γ2 (*PPAR-γ2*) has been convincingly associated with insulin resistance (IR) and type 2 diabetes (T2D) among Europeans, in interaction with a high-fat diet. Mexico is disproportionally affected by obesity and T2D however, whether the Pro12Ala polymorphism is associated with early metabolic complications in this population is unknown. We assessed the association of *PPAR-γ2* Pro12Ala with metabolic traits in 1457 Mexican children using linear regression models. Interactions between *PPAR-γ2* Pro12Ala and circulating lipids on metabolic traits were determined by adding an interaction term to regression models. We observed a high prevalence of overweight/obesity (49.2%), dyslipidemia (34.9%) and IR (11.1%). We detected nominally significant/significant interactions between lipids (total cholesterol, HDL-cholesterol, LDL-cholesterol), the *PPAR-γ2* Pro12Ala genotype and waist-to-hip ratio, fasting insulin, HOMA-IR and IR (9.30 × 10^−4^  ≤ P_interaction_ ≤ 0.04). Post-hoc subgroup analyses evidenced that the association between the *PPAR-γ2* Pro12Ala genotype and fasting insulin, HOMA-IR and IR was restricted to children with total cholesterol or LDL-cholesterol values higher than the median (0.02 ≤ P ≤ 0.03). Our data support an association of the Pro12Ala polymorphism with IR in Mexican children and suggest that this relationship is modified by dyslipidemia.

Peroxisome proliferator-activated receptor-γ2 (PPAR-γ2) is a ligand activated transcription factor highly expressed in adipose tissue and is intimately involved in the regulation of adipogenesis, glucose and lipid homeostasis and insulin sensitivity^1^. PPARγ is the molecular target of the anti-diabetic drug thiazolidinedione (TZD)^1^. A missense coding variant in *PPAR-γ2* resulting in a proline to alanine substitution (Pro12Ala, rs1801282) has been associated with a 30–50% decrease in ligand-induced activity^2^.

The association of *PPAR-γ2* Pro12Ala polymorphism with type 2 diabetes (T2D) is well established. A recent literature-based candidate gene meta-analysis by Gouda *et al*. in 32,849 T2D cases and 47,456 controls from Europe, North America and East Asia determined that the deleterious Pro allele is associated with a 16% increased risk of T2D^3^. More recently, a large-scale association study combining the data from GWAS and from the custom array Metabochip in 34,840 T2D cases and 114,981 controls predominantly of European descent confirmed that the deleterious Pro12 allele was associated with a 13% increased risk of T2D^4^. The association of *PPAR-γ2* Pro12Ala polymorphism with body mass index (BMI) has been long debated in literature, but a recent meta-analysis of 49,092 subjects from diverse ethnic backgrounds demonstrated that the *PPAR-γ2* Pro12 allele was associated with a lower BMI^5^. The authors also evidence a trend for a stronger effect of the Pro12 allele in individuals of European ancestry^5^.

Dietary fats are known ligands for PPAR-γ2 and have been shown to interact with the Pro12Ala polymorphism to modulate obesity-related traits in six independent studies^6^,^7^,^8^,^9^,^10^,^11^. Similar gene x diet interactions have been described between dietary fat intake and Pro12Ala polymorphism for insulin resistance (IR) and T2D-related traits^12^,^13^. These results are suggestive of a diet-dependent interaction between the Pro12Ala polymorphism, body weight and T2D that can possibly explain the conflicting results regarding the influence of this variant on metabolic traits in individual studies.

The Mexican population is disproportionately affected by both obesity and T2D. In 2008, the United Nations Food and Agricultural Organization estimated the prevalence of obesity in Mexico to be 32.8%, surpassing that of the United States^14^; the prevalence of T2D in Mexico is estimated to be as high as 14.4%^15^. According to the Mexican National Institute of Public Health, 34.4% of children between 5 and 11 years of age were overweight or obese in 2011^16^. This is especially problematic given that childhood obesity is the main predictor of adult obesity^17^.

Despite the well-established association between the *PPAR-γ2* Pro12Ala variant, obesity and T2D in populations of European ancestry and the high prevalence of these conditions in Mexicans, only a few studies have examined these associations in a Mexican population. The *PPAR-γ2* Ala12 allele has been associated with a higher risk of overweight/obesity in adult Mexican Mestizo subjects and in five Mexican Amerindian groups^18^. This trend was confirmed in 921 Mexican-American adults from the San Antonio Family Heart Study, where carriers of at least one Ala allele had a higher BMI and waist circumference^19^. No associations between the *PPAR-γ2* Pro12Ala polymorphism and T2D were observed in three modestly powered studies of Mexican adults^20^,^21^,^22^. In 473 adult individuals from 89 Mexican-American families, the *PPAR-γ2* Pro12Ala polymorphism was not associated with IR measured by oral and intravenous glucose tolerance tests^23^. To our knowledge, the association of Pro12Ala with obesity/T2D related traits has never been examined in Mexican children. Thus, we aimed to determine the association between the *PPAR-γ2* Pro12Ala variant and metabolic parameters in 1457 Mexican children and its interaction with circulating lipids used as stable surrogate of a high-fat diet.

## Results

### Phenotypic characteristics of the studied population

Anthropometric and biochemical characteristics of the study population are presented in Table 1. Of the 1457 children sampled (between 6 and 14 years old, average age 9.24 ± 2.07), 1.4% of the children in the population were underweight, 49.4% were a normal weight, 21.3% were overweight and 27.9% were obese. Insulin resistance was identified in 11.1% of children. 3.1% of children had IFG and only one child was diabetic. Hypertension was present in 22 children (1.5%). Dyslipidemia was identified in 34.9% of the population. Children displayed a significantly higher BMI in the Cuauhtémoc area (20.71 ± 4.34) than in the other areas (Independencia: 19.60 ± 4.15; Nezahualcóyotl: 19.15 ± 4.07; Morelos: 19.32 ± 4.11) using a one-way ANOVA and a Tukey post-hoc test (P between 2.1 × 10^−6^ and 4.3 × 10^−3^, data not shown). The genotype distribution of *PPAR-γ2* Pro12Ala in the study population was 73.9% (n = 1067), 24.5% (n = 354) and 1.6% (n = 23) for the Pro/Pro, Pro/Ala and Ala/Ala genotypes, respectively. Thirteen individuals were not successfully genotyped (Pro12Ala genotyping call rate: 99.1%).

### Associations/interactions between *PPAR-γ2* Pro12Ala and metabolic quantitative traits

Knowing that previous reports provide evidence for interactions between *PPAR-γ2* Pro12Ala and dietary exposures to alter metabolic traits and that *PPAR-γ2* is activated by dietary lipids, we tested the interaction between *PPAR-γ2* Pro12Ala and fasting plasma lipid concentrations on metabolic traits^24^,^25^. Circulating plasma lipids were used as stable surrogate of a high-fat diet^26^. A nominally significant interaction between *PPAR-γ2* Pro12Ala and HDL-C was found to modulate WHR (main genotype effect: β = −0.57 ± 0.20, p = 4.89 × 10^−3^; interaction: β = 1.14 × 10^−2^ ± 3.81 × 10^−3^, p = 2.91 × 10^−3^) (Table 2). Nominally significant interactions between *PPAR-γ2* Pro12Ala and TC on fasting insulin levels (main genotype effect: β = 0.55 ± 0.26, p = 0.04; interaction: β = −3.79 × 10^−3^ ± 1.62 × 10^−3^, p = 0.02) and HOMA-IR (main genotype effect: β = 0.49 ± 0.26, p = 0.06; interaction: β = −3.38 × 10^−3^ ± 1.61 × 10^−3^, p = 0.04) were also identified. Given the interactions between plasma lipids, insulin and HOMA-IR, we subsequently tested the interaction between circulating lipids on the presence of IR (Table 3). Both TC and plasma LDL-C concentrations were found to interact with *PPAR-γ2* Pro12Ala to influence the presence of IR (OR_main genetic effect_ = 18.39, 95% CI 2.57–131.79, OR _interaction_ = 0.98, 95% CI 0.97–0.99, p_main genetic effect_ = 9.54 × 10^−4^, p_interaction_ = 9.30 × 10^−4^; OR_main genetic effect_ = 8.70, 95% CI 1.62–46.87, OR_interaction_ = 0.98, 95% CI 0.96–0.99, p_main genetic effect_ = 0.01, p _interaction_ = 8.09 × 10^−3^, respectively).

We further investigated the direction of the genetic effects of the *PPAR-γ2* Pro12Ala polymorphism on adiposity and insulin resistance parameters showing interaction with lipids. Genetic association tests in subgroups were performed using the median of plasma lipids to classify the population into high and low groups (Table 4). Despite a nominally significant interaction between *PPAR-γ2* Pro12Ala and HDL-C on WHR, the results failed to reach significance in the subgroup analyses. In the high TC subgroup, the carriers of Ala12 displayed nominally significant lower fasting insulin levels/HOMA-IR values (β = −0.19 ± 0.08, p = 0.02 and β = −0.17 ± 0.08, p = 0.03, respectively). No evidence of association between *PPAR-γ2* Pro12Ala, fasting insulin levels and HOMA-IR was observed in the low TC subgroup (p = 0.24 for both). When LDL-C and TC levels were high, Ala12 carriers were also found to have a nominally significant reduced risk of developing IR (OR = 0.44, 95% CI 0.27–0.87, p = 0.02 and OR = 0.41, 95% 0.20–0.84, p = 0.02, respectively). No association between *PPAR-γ2* Pro12Ala and IR was found in the low LDL-C and TC groups (p = 0.07 for both).

## Discussion

In the present study we examined the association of the Pro12Ala variant in *PPAR-γ2* with metabolic traits and identified nominally significant or significant evidence for gene-environment interactions involving *PPAR-γ2* genotype and high circulating concentrations of TC, HDL-C and LDL-C influencing WHR, plasma insulin, HOMA-IR and IR.

We observed a high prevalence of obesity, IR and dyslipidemia in our sample of 1457 Mexican children. Mexico is experiencing significant epidemiological transitions. Reduced physical activity due to urbanization and technological innovations and shifts in dietary patterns away from traditional high-fiber foods to the increased consumption of processed foods laden with fat, refined carbohydrates and added sugar have resulted in a rise in non-communicable chronic diseases among all age groups^27^. Indeed, the prevalence of overweight and obesity in Mexican children reached 34.4% in 2011, representing one of the highest rates of pediatric obesity in the world^16^. Our sample exceeds the national average with a prevalence of overweight/obesity of 49.2%, which may be partly explained by our strategy to recruit children within an urban setting.

Pediatric obesity is accompanied by an early onset of a number of co-morbidities including T2D, hypertension, dyslipidemia, and non-alcoholic fatty liver disease^28^. The prevalence of dyslipidemia in our sample was an outstanding 34.9%, much higher than previously reported. The high prevalence of dyslipidemia may be attributed to a diet rich in refined carbohydrates and animal fats but limited in fiber^29^. Furthermore, we cannot exclude the possibility that the prevalence of dyslipidemia reported in this study may stem from the employed definition. Abnormal concentrations of one or two lipids are routinely used to identify dyslipidemia. However, the use of three lipids in our study may have artificially increased the prevalence of dyslipidemia in our sample. The prevalence of IR in our sample (11%) is lower than previously reported. In a cross-sectional study of Mexican children aged 7–18, the prevalence of IR was estimated at 20.3% while the National Health and Nutrition Examination Survey found 52.1% of obese Mexican-Americans aged 12–19 to have IR (compared to 23.4% of obese children in our sample, data not shown)^30^,^31^. This discrepancy may be attributed to the younger age of our sample given that insulin and glucose concentrations gradually increase with age^32^. We also observed a very low prevalence of hypertension in our sample (1.5%). Previous reports show the prevalence of hypertension among Mexican children varying from 4.7% to 14%^33^,^34^,^35^. These studies however classified hypertension using percentiles rather than a threshold, making comparisons challenging.

Since its discovery, the *PPAR-γ2* Pro12Ala polymorphism has garnered considerable interest due to its ability to modulate both T2D and obesity risk. Results from GWAS in diverse ethnic groups have established the protective role of the Ala12 allele against T2D despite it being an obesity-risk allele, as suggested by a recent large-scale meta-analysis^4^,^5^. Allele frequencies of the Pro12Ala polymorphism vary among ethnic groups with the highest Ala12 allele frequencies generally reported in Caucasian, South Asian and South American (all 12%) populations in the 1000 Genomes Project. The lowest frequencies are found among East Asian (3%) or African (0.5%) populations. In our sample, the frequency of the Ala12 allele was similar to the allele frequencies reported in the 1000 Genomes Project for Mexican-American adults (14% vs 13%, respectively). In addition to many other genetic variants, the varying frequency of the Pro12Ala polymorphism among ethnic groups contributes to the contrasting patterns of predisposition to obesity and T2D among populations.

Fatty acids, in particular unsaturated fatty acids, serve as ligands for *PPAR-γ2*. Therefore, we examined the interaction between circulating lipids as a surrogate for a high-fat diet and *PPAR-γ2* genotype on metabolic traits^36^. Previous studies have shown diet-gene interactions between total, saturated or polyunsaturated fat intake on obesity and T2D related traits, however to our knowledge, ours is the first study to report significant interactions between *PPAR-γ2* genotype and circulating lipids on IR. IR is driven by dyslipidemia (elevated concentrations of TC and LDL-C and decreased concentrations of HDL-C) and is a strong predictor of T2D^37^. A nominal association towards lower fasting insulin concentration and lower HOMA-IR was observed among carriers of the Ala12 allele when TC levels were high. Carriers of the Ala12 allele were found to have a decreased risk for IR despite high circulating LDL-C, further suggesting the protective role of the Ala12 allele against the development of IR amid dyslipidemia^38^. In our population, a nominally significant interaction between *PPAR-γ2* genotype and HDL-C on WHR was identified with a trend towards low WHR in carriers of the Ala12 allele. The well-established inverse relationship between circulating HDL-C and abdominal obesity was not observed in the subgroup of carriers of the Ala12 allele with high HDL-C concentrations^39^. This finding warrants further replication in another independent population of Mexican children.

These results must be interpreted with consideration for the acknowledged limitations. Firstly, our population cannot be considered representative of the Mexican pediatric population as a whole as the prevalence of overweight and obesity in Mexico is higher in urban areas with greater economic development (i.e. northern Mexico and Mexico City)^40^. Therefore, our population is representative of the urban population of central Mexico as the recruitment was random. The Mexican population is admixed with Native American (65%), European (30%), and West African ancestries (5%) with proportions being affected by geographic, demographic and historical factors^41^. As such, genetic heterogeneity exists between and within different regions of Mexico. Although all of the children in our study reside in Mexico City, we did not have access to ancestry-informative markers and thus could not adjust for genetic admixture Circulating lipid levels were used as a surrogate for a high- fat diet, however this assumption could not be confirmed as dietary intake was not directly measured. We acknowledge that our power was modest, especially considering the Ala12Ala genotype (N = 23) and therefore our findings deserve further investigation. Due to our modest sample size, most of our results did not reach statistical significance after adjusting for multiple testing with Bonferroni correction (P < 2.08 × 10^−3^) and warrant replication in independent Mexican pediatric populations (Supplementary Table S1). Lastly, due to the cross-sectional nature of this study, causality cannot be inferred.

The results of the current study are noteworthy because the association between *PPAR-γ2* Pro12Ala and obesity and T2D-related traits has never been examined in Mexican children. This is the first study to our knowledge to report a significant gene-environment interaction between *PPAR-γ2* Pro12Ala, circulating lipids and markers of IR in a pediatric Mexican population. Mexican children are a high-risk population for obesity and metabolic complications and the prevalence of these conditions will likely dramatically increase in this population as they age. Our results also show that genetic predisposition can alter metabolic traits early in life in presence of an obesogenic environment. Taken together, the present study demonstrates the urgency of preventing and treating obesity and T2D and presents childhood as a critical period of opportunity for prevention and intervention strategies. These results also highlight the need for a comprehensive understanding of the genetics of obesity and T2D in diverse ethnic groups in order to establish personalized/ stratified intervention strategies.

In conclusion, our data show an association of the Pro12Ala allele with IR in a sample of 1457 Mexican children. Our results also suggest an interaction between *PPAR-γ2* Pro12Ala genotype and circulating lipids on IR. Knowing that Mexican children are at high risk for obesity and T2D, *PPAR-γ2* genotype could be used in conjunction with other known obesity and T2D genes to guide early prevention strategies in the management of these diseases.

## Methods

### Study population

A total of 1457 unrelated children aged 6–14 were randomly selected to participate in a cross-sectional study from four areas in Mexico City at the Primary Care Unit of the National Mexican Social Security Institute (Cuauhtémoc West, Independencia South, Nezahualcóyotl Est and Morelos North area). Recruitment was done in collaboration with local public schools. The study started in July 2011 and is still ongoing. Children who had diagnosis of infectious disease, gastrointestinal disorders, administration of antimicrobial agents (within 6 months previous to study), incomplete questionnaires or biological samples were excluded. The study protocol was approved by the Mexican Social Security Institute National Committee and the Ethical Committee Board and informed consent was obtained from both parents and the child, in accordance with the Declaration of Helsinki.

### Phenotyping

All participants were weighed using a digital scale (Seca, Hamburg, Germany) and height was measured with a portable stadiometer (Seca 225, Hamburg, Germany). Waist circumference was measured at the midpoint between the lowest rib and the iliac crest after a normal exhalation with children in the standing position. Hip circumference was measured at the level of the greater trochanters. Body mass index was calculated as weight (kg)/height (m)^2^ and classified (underweight, normal weight, overweight, obese) according to the Centers for Disease Control and Prevention CDC 2000 references. Blood samples were obtained following an 8–12 hour fast and were analyzed for fasting glucose, total cholesterol (TC), high-density lipoprotein cholesterol (HDL-C) and low-density lipoprotein cholesterol (LDL-C) and triglycerides (TG) using the ILab 350 Clinical Chemistry System (Instrumentation Laboratory IL. Barcelona Spain). Insulin (IU) was measured by chemiluminescence (IMMULITE, Siemens, USA) and homeostatic model assessment of insulin resistance (HOMA-IR) was calculated using the equation by Matthews *et al* 42. Due to the risk of blood hemolysis, fasting insulin values <1 μU/mL were discarded from the study. Insulin resistance was defined as HOMA-IR ≥3.4 (the 90^th^ percentile of HOMA-IR in a population of healthy Mexican children)^43^. Hypertension was defined as average measured blood pressure above the American Heart Association’s recommendations (systolic ≥140 mmHg or diastolic ≥90 mmHg). Dyslipidemia was defined as fasting TG ≥ 100  mg/dL (0–9 years of age) or TG ≥ 130 mg/dL (10–19 years of age) and/or HDL-C <35 mg/dL and/or LDL-C ≥130 mg/dL, according to current recommendations^44^,^45^.

### Genotyping

Genomic DNA was isolated from peripheral blood using a standard extraction protocol on an Autogen FLEX STAR (Holliston, Massachusetts USA). Genotyping of the Pro12Ala polymorphism was performed using the TaqMan Open Array Real-Time PCR System (Life Technologies, Carlsbad, USA), following the manufacturer’s instructions. The Open Array experiment involved 64 polymorphisms. From the initial sample of 1559 participants, 102 were excluded from the current analysis because i) no blood sample was collected for DNA extraction; ii) DNA extraction was unsuccessful; iii) the genotyping success rate of the Open Array experiment based on the 64 polymorphisms was < 90.6% (≥6 genotypes missing). The current analysis included 1457 children. The Pro12Ala genotyping call rate was 99.1%. Deviation from Hardy-Weinberg equilibrium (HWE) for Pro12Ala was tested using a chi-square test and no deviation from HWE was observed (p = 0.30).

### Statistical analysis

The normal distribution of continuous variables was tested using the Shapiro-Wilk test. All traits of interest deviated significantly from normality. Inverse normal transformations corrected the lack of normality for BMI, WHR, insulin, HOMA-IR, and TG (Supplementary Figure S1). Non-biological outlier data were discarded. The effect of the rs1801282 variant on metabolic traits (BMI, WHR, fasting glucose, fasting insulin, HOMA-IR, TC, TG, HDL-C and LDL-C) was determined under an additive genetic model using linear regression adjusted for age, sex and recruitment center. The minor allele Ala12 was considered as the effect allele. Interactions between plasma lipids (as continuous traits) and Pro12Ala on metabolic traits were investigated by adding an interaction term to the linear regression model. To investigate further significant interactions, genetic association tests in subgroups were performed using the median of the interacting factor to classify the population into high and low groups. Differences between recruitment centers were determined using a one-way ANOVA and a Tukey post-hoc test. After adjusting for multiple testing using Bonferroni correction (6 metabolic traits in interaction with 4 lipid traits), a p-value below 2.08 × 10^−3^ (0.05/24) was considered statistically significant and a p-value between 0.05 and 2.08 × 10^−3^ was considered nominally significant. All statistical analyses were performed using SPSS software (version 20.0). We assessed the power of our sample using QUANTO software version 1.2.4 (University of Southern California, Los Angeles, CA, USA).

## Additional Information

**How to cite this article**: Stryjecki, C. *et al*. Association between *PPAR-γ2* Pro12Ala genotype and insulin resistance is modified by circulating lipids in Mexican children. *Sci. Rep*. **6**, 24472; doi: 10.1038/srep24472 (2016).

## Supplementary Material

Supplementary Information

## Figures and Tables

**Table 1:** General characteristics of the studied population of Mexican children by *PPAR-γ2* Pro12Ala genotype.

Characteristics	N = 1457	Pro12Pro (N = 1067)	Pro12Ala (N = 354)	Ala12Ala (N = 23)
Male/ Female, N	771/686	565/502	190/164	8/15
Age	9.24 ± 2.07	9.27 ± 2.05	9.19 ± 2.15	8.91 ± 1.73
Waist Circumference (cm)	66.47 ± 11.78	66.57 ± 11.71	66.67 ± 12.11	62.51 ± 8.84
WHR	0.85 ± 0.06	0.85 ± 0.06	0.85 ± 0.06	0.85 ± 0.05
BMI (kg/m^2^)	19.65 ± 4.20	19.67 ± 4.17	19.76 ± 4.34	18.33 ± 3.37
Systolic blood pressure (mmHg)	98.57 ± 10.86	98.45 ± 11.03	99.00 ± 10.51	97.70 ± 8.83
Diastolic blood pressure (mmHg)	66.24 ± 8.80	66.03 ± 8.96	66.96 ± 8.23	65.09 ± 9.53
Glucose (mmol/L)	4.57 ± 0.53	4.56 ± 0.53	4.57 ± 0.51	4.65 ± 0.61
Insulin (μU/mL)	8.68 ± 7.10	9.15 ± 7.06	9.12 ± 7.18	7.08 ± 4.05
HOMA-IR	1.87 ± 1.52	1.88 ± 1.52	1.88 ± 1.55	1.42 ± 0.78
TG (mg/dL)	93.62 ± 49.70	94.77 ± 50.37	90.72 ± 48.00	90.78 ± 45.62
TC (mg/dL)	157.25 ± 33.56	157.21 ± 33.98	156.91 ± 31.87	166.43 ± 42.27
HDL-C (mg/dL)	50.60 ± 12.82	50.25 ± 12.59	51.41 ± 13.42	52.65 ± 15.25
LDL-C (mg/dL)	102.39 ± 26.42	102.78 ± 27.22	101.35 ± 23.93	102.30 ± 28.51
Insulin Resistance, N (%)	127 (11.1%)	97 (11.4%)	28 (10.5%)	1 (6.7%)
Dyslipidemia, N (%)	509 (34.9%)	385 (36.1%)	113 (31.9%)	9 (39.1%)
Hypertension, N (%)	22 (1.5%)	19 (1.8%)	3 (0.9%)	0 (0%)
Hyperglycemia, N (%)	45 (3.1%)	35 (3.3%)	8 (2.3%)	1 (4.3%)

Abbreviations: BMI, body mass index; HDL-C, high-density lipoprotein cholesterol; HOMA-IR, homeostatic model assessment of insulin resistance; LDL-C, low-density lipoprotein cholesterol; TC, total cholesterol; TG, triglycerides; T2D, type 2 diabetes; WHR, waist-to-hip ratio.

Data are means ± standard deviation.

**Table 2:** Interactions between circulating lipids, *PPAR-γ2* Pro12Ala and metabolic quantitative traits.

Outcome	Pro12Ala x TC		Pro12Ala x TG^a^		Pro12Ala x HDL-C		Pro12Ala x LDL-C	
	Main Genetic Effect	Interaction	Main Genetic Effect	Interaction	Main Genetic Effect	Interaction	Main Genetic Effect	Interaction
BMI^a^	0.16 ± 0.23 (0.49)	−1.13 × 10^−3^ ± 1.42 × 10^−3^ (0.42)	0.01 ± 0.04 (0.85)	0.01 ± 0.05 (0.85)	−0.21 ± 0.18 (0.27)	4.10 × 10^−3^ ± 3.48 × 10^−3^ (0.24)	0.08 ± 0.20 (0.68)	−8.94 × 10^−4^ ± 1.90 × 10^−3^ (0.64)
WHR^a^	−0.05 ± 0.24 (0.85)	2.77 × 10^−4^ ± 1.50 × 10^−3^ (0.85)	0.02 ± 0.05 (0.70)	0.01 ± 0.05 (0.84)	**−0.57 ± 0.20 (4.89 × 10^−3^)**	**1.14 × 10^−2^ ± 3.81 × 10^−3^(2.91 × 10^−3^)**	0.08 ± 0.21 (0.69)	−7.22 × 10^−4^ ± 2.03 × 10^−3^ (0.72)
Glucose	−0.03 ± 0.12 (0.83)	2.51 × 10^−4^ ± 7.44 × 10^−4^ (0.74)	0.02 ± 0.03 (0.37)	0.03 ± 0.03 (0.20)	0.03 ± 0.10 (0.78)	−3.85 × 10^−4^ ± 1.97 × 10^−3^ (0.84)	0.02 ± 0.11 (0.86)	3.65 × 10^−5^ ± 1.03 × 10^−3^ (0.97)
Insulin^a^	0.55 ± 0.26 (0.04)	−3.79 × 10^−3^ ± 1.62 × 10^−3^ (0.02)	−0.04 ± 0.05 (0.45)	−0.05 ± 0.05 (0.36)	−0.06 ± 0.21 (0.77)	6.51 × 10^−4^ ± 3.91 × 10^−3^ (0.77)	0.25 ± 0.23 (0.27)	−2.93 × 10^−3^ ± 2.16 × 10^−3^ (0.18)
HOMA-IR^a^	0.49 ± 0.26 (0.06)	−3.38 × 10^−3^ ± 1.61 × 10^−3^ (0.04)	−0.03 ± 0.05 (0.58)	−0.04 ± 0.05 (0.50)	−0.04 ± 0.21 (0.86)	3.59 × 10^−4^ ± 3.94 × 10^−3^ (0.93)	0.21 ± 0.23 (0.36)	−2.40 × 10^−3^ ± 2.15 × 10^−3^ (0.26)

Abbreviations: BMI, body mass index; HDL-C, high-density lipoprotein cholesterol; HOMA-IR, homeostatic model assessment of insulin resistance; LDL-C, low-density lipoprotein cholesterol; SE, standard error; TC, total cholesterol; TG, triglycerides; WC, waist circumference; WHR, waist-to-hip ratio.

Data presented are β ± SE (p _value_). All models were adjusted for age, sex, and recruitment center. Values in bold indicate nominally significant or significant main genetic effects and interactions (p < 0.05).

^a^Inverse normal transformed variables.

**Table 3:** Interactions between circulating lipids, *PPAR-γ2* Pro12Ala and the presence of insulin resistance.

	OR_interaction_ (95% CI)	P _interaction_	OR_main genetic effect_ (95% CI)	P _main genetic effect_
PPARγ x TC	**0.98 (0.97–0.99)**	**9.30 × 10**^**−4**^	**18.39 (2.57–131.79)**	**9.54 × 10**^**−4**^
PPARγ x TG^a^	1.06 (0.61–1.85)	0.84	0.87 (0.47–1.61)	0.66
PPARγ x HDL-C	0.98 (0.94–1.02)	0.28	2.52 (0.39–16.43)	0.33
PPARγ x LDL-C	**0.98 (0.96–0.99)**	**8.09 × 10**^**−3**^	**8.70 (1.62–46.87)**	**0.01**

Abbreviations: CI, confidence interval; HDL-C, high-density lipoprotein cholesterol; LDL-C, low-density lipoprotein cholesterol; OR, odds ratio; TC, total cholesterol; TG, triglycerides.

Data presented are OR (95% CI). All models were adjusted for age, sex, and recruitment center. Values in bold indicate nominally significant or significant main genetic effects and interactions (p < 0.05).

^a^Inverse normal transformed variables.

**Table 4:** Circulating lipid subgroup analysis for significant interactions between *PPAR-γ2* Pro12Ala and metabolic traits.

	β ± SE	p_value_
	WHR^a^	
Low HDL-C	−0.10 ± 0.08	0.17
High HDL-C	0.09 ± 0.07	0.20
	Insulin^a^	
Low TC	0.09 ± 0.08	0.24
High TC	−0.19 ± 0.08	**0.02**
	HOMA-IR^a^	
Low TC	0.10 ± 0.08	0.24
High TC	−0.17 ± 0.08	**0.03**
	OR (95% CI)	p _value_
	Insulin Resistance	
Low TC	1.69 (0.92–2.96)	0.07
High TC	0.41 (0.20–0.84)	**0.02**
Low LDL-C	1.72 (0.97–3.04)	0.07
High LDL-C	0.44 (0.27–0.87)	**0.02**

Abbreviations: CI, confidence interval; HDL-C, high-density lipoprotein cholesterol; LDL-C, low-density lipoprotein cholesterol; OR, odds ratio; TC, total cholesterol; WHR, waist-to-hip ratio. Data presented are β ± SE (p _value_) or OR (95% CI). All models were adjusted for age, sex, and recruitment center. Values in bold indicate nominally significant or significant interactions (p < 0.05).

^a^Inverse normal transformed variables.

## References

[b1] Qi Q. & Hu F. B. Genetics of type 2 diabetes in European populations. J. Diabetes 4, 203–212, doi: 10.1111/j.1753-0407.2012.00224.x (2012). 22781158 PMC3422419

[b2] Deeb S. S. . A Pro12Ala substitution in PPARgamma2 associated with decreased receptor activity, lower body mass index and improved insulin sensitivity. Nat. Genet. 20, 284–287 (1998). 9806549 10.1038/3099

[b3] Gouda H. N. . The association between the peroxisome proliferator-activated receptor-gamma2 (PPARG2) Pro12Ala gene variant and type 2 diabetes mellitus: a HuGE review and meta-analysis. Am. J. Epidemiol. 171, 645–655, doi: 10.1093/aje/kwp450 (2010). 20179158 PMC2834889

[b4] Morris A. P. . Large-scale association analysis provides insights into the genetic architecture and pathophysiology of type 2 diabetes. Nat. Genet. 44, 981–990, doi: 10.1038/ng.2383 (2012). 22885922 PMC3442244

[b5] Galbete C. . Pro12Ala variant of the PPARG2 gene increases body mass index: An updated meta-analysis encompassing 49,092 subjects. Obesity (Silver Spring) 21, 1486–1495, doi: 10.1002/oby.20150 (2013). 23666678

[b6] Robitaille J., Despres J. P., Perusse L. & Vohl M. C. The PPAR-gamma P12A polymorphism modulates the relationship between dietary fat intake and components of the metabolic syndrome: results from the Quebec Family Study. Clin. Genet. 63, 109–116 (2003). 12630956 10.1034/j.1399-0004.2003.00026.x

[b7] Garaulet M., Smith C. E., Hernandez-Gonzalez T., Lee Y. C. & Ordovas J. M. PPARgamma Pro12Ala interacts with fat intake for obesity and weight loss in a behavioural treatment based on the Mediterranean diet. Mol. Nutr. Food Res. 55, 1771–1779, doi: 10.1002/mnfr.201100437 (2011). 22102511 PMC3951915

[b8] Luan J. . Evidence for gene-nutrient interaction at the PPARgamma locus. Diabetes 50, 686–689 (2001). 11246892 10.2337/diabetes.50.3.686

[b9] Heikkinen S. . The Pro12Ala PPARgamma2 variant determines metabolism at the gene-environment interface. Cell Metab. 9, 88–98, doi: 10.1016/j.cmet.2008.11.007 (2009). 19117549

[b10] Lamri A. . Dietary fat intake and polymorphisms at the PPARG locus modulate BMI and type 2 diabetes risk in the D.E.S.I.R. prospective study. Int. J. Obes. (Lond.) 36, 218–224, doi: 10.1038/ijo.2011.91 (2012). 21540831

[b11] Razquin C. . The Mediterranean diet protects against waist circumference enlargement in 12Ala carriers for the PPARgamma gene: 2 years’ follow-up of 774 subjects at high cardiovascular risk. Br. J. Nutr. 102, 672–679, doi: 10.1017/S0007114509289008 (2009). 19267951

[b12] Ylonen S. K. . The Pro12Ala polymorphism of the PPAR-gamma2 gene affects associations of fish intake and marine n-3 fatty acids with glucose metabolism. Eur. J. Clin. Nutr. 62, 1432–1439, doi: 10.1038/sj.ejcn.1602882 (2008). 17700648

[b13] Soriguer F. . Pro12Ala polymorphism of the PPARG2 gene is associated with type 2 diabetes mellitus and peripheral insulin sensitivity in a population with a high intake of oleic acid. J. Nutr. 136, 2325–2330 (2006). 16920849 10.1093/jn/136.9.2325

[b14] Food and Agriculture Organization of the United Nations, The state of food and agriculture. (2013) Available at: http://www.fao.org/docrep/018/i3300e/i3300e00.htm. (Accessed: 4th April 2015).

[b15] Reynoso-Noveron N. . Estimated incidence of cardiovascular complications related to type 2 diabetes in Mexico using the UKPDS outcome model and a population-based survey. Cardiovasc. Diabetol. 10, 1, doi: 10.1186/1475-2840-10-1 (2011). 21214916 PMC3023678

[b16] Gutiérrez J. P. . Encuesta Nacional de Salud y Nutrición 2012. Resultados Nacionales. Cuernavaca, México: Instituto Nacional de Salud Pública. (2012).

[b17] Parsons T. J., Power C., Logan S. & Summerbell C. D. Childhood predictors of adult obesity: a systematic review. Int. J. Obes. Relat. Metab. Disord. 23 Suppl 8, S1–107 (1999). 10641588

[b18] Canizales-Quinteros S. . Association of PPARG2 Pro12Ala variant with larger body mass index in Mestizo and Amerindian populations of Mexico. Hum. Biol. 79, 111–119 (2007). 17985660 10.1353/hub.2007.0022

[b19] Cole S. A. . The Pro12Ala variant of peroxisome proliferator-activated receptor-gamma2 (PPAR-gamma2) is associated with measures of obesity in Mexican Americans. Int. J. Obes. Relat. Metab. Disord. 24, 522–524 (2000). 10805513 10.1038/sj.ijo.0801210

[b20] Martinez-Gomez L. E. . A replication study of the IRS1, CAPN10, TCF7L2, and PPARG gene polymorphisms associated with type 2 diabetes in two different populations of Mexico. Ann. Hum. Genet. 75, 612–620, doi: 10.1111/j.1469-1809.2011.00668.x (2011). 21834909

[b21] Gamboa-Melendez M. A. . Contribution of common genetic variation to the risk of type 2 diabetes in the Mexican Mestizo population. Diabetes 61, 3314–3321, doi: 10.2337/db11-0550 (2012). 22923468 PMC3501881

[b22] Cruz M. . Candidate gene association study conditioning on individual ancestry in patients with type 2 diabetes and metabolic syndrome from Mexico City. Diabetes Metab. Res. Rev. 26, 261–270, doi: 10.1002/dmrr.1082 (2010). 20503258

[b23] Black M. H. . Evidence of interaction between PPARG2 and HNF4A contributing to variation in insulin sensitivity in Mexican Americans. Diabetes 57, 1048–1056, doi: 10.2337/db07-0848 (2008). 18162503 PMC4447520

[b24] Vidal-Puig A. . Regulation of PPAR gamma gene expression by nutrition and obesity in rodents. J. Clin. Invest. 97, 2553–2561, doi: 10.1172/JCI118703 (1996). 8647948 PMC507341

[b25] Vidal-Puig A. J. . Peroxisome proliferator-activated receptor gene expression in human tissues. Effects of obesity, weight loss, and regulation by insulin and glucocorticoids. J. Clin. Invest. 99, 2416–2422, doi: 10.1172/JCI119424 (1997). 9153284 PMC508081

[b26] Hodson L., Skeaff C. M. & Fielding B. A. Fatty acid composition of adipose tissue and blood in humans and its use as a biomarker of dietary intake. Prog. Lipid Res. 47, 348–380, doi: 10.1016/j.plipres.2008.03.003 (2008). 18435934

[b27] Rivera J. A., Barquera S., Gonzalez-Cossio T., Olaiz G. & Sepulveda J. Nutrition transition in Mexico and in other Latin American countries. Nutr. Rev. 62, S149–157 (2004). 15387482 10.1111/j.1753-4887.2004.tb00086.x

[b28] Daniels S. R., Jacobson M. S., McCrindle B. W., Eckel R. H. & Sanner B. M. American Heart Association Childhood Obesity Research Summit Report. Circulation 119, e489–517, doi: 10.1161/CIRCULATIONAHA.109.192216 (2009). 19332458

[b29] Jenkins D. J. . Effects of a dietary portfolio of cholesterol-lowering foods vs lovastatin on serum lipids and C-reactive protein. JAMA 290, 502–510, doi: 10.1001/jama.290.4.502 (2003). 12876093

[b30] Romero-Polvo A. . Association between dietary patterns and insulin resistance in Mexican children and adolescents. Ann. Nutr. Metab. 61, 142–150, doi: 10.1159/000341493 (2012). 23037180

[b31] Lee J. M., Okumura M. J., Davis M. M., Herman W. H. & Gurney J. G. Prevalence and determinants of insulin resistance among U.S. adolescents: a population-based study. Diabetes Care 29, 2427–2432, doi: 10.2337/dc06-0709 (2006). 17065679

[b32] Aguirre-Arenas J., Escobar-Perez M. & Chavez-Villasana A. [Evaluation of food consumption patterns and nutrition in 4 rural communities]. Salud Publica Mex. 40, 398–407 (1998). 9842277

[b33] Juarez-Rojas J. G. . Blood pressure and associated cardiovascular risk factors in adolescents of Mexico City. Arch Cardiol. Mex. 78, 384–391 (2008). 19205546

[b34] Dyson P. A. . High rates of child hypertension associated with obesity: a community survey in China, India and Mexico. Paediatr Int Child Health 34, 43–49, doi: 10.1179/2046905513Y.0000000079 (2014). 24091383

[b35] Ramos-Arellano L. E. . Body fat distribution and its association with hypertension in a sample of Mexican children. J. Investig. Med. 59, 1116–1120, doi: 10.231/JIM.0b013e31822a29e1 (2011). 21804403

[b36] Xu H. E. . Molecular recognition of fatty acids by peroxisome proliferator-activated receptors. Mol. Cell 3, 397–403 (1999). 10198642 10.1016/s1097-2765(00)80467-0

[b37] McLaughlin T. . Use of metabolic markers to identify overweight individuals who are insulin resistant. Ann. Intern. Med. 139, 802–809 (2003). 14623617 10.7326/0003-4819-139-10-200311180-00007

[b38] Kubota N. . PPAR gamma mediates high-fat diet-induced adipocyte hypertrophy and insulin resistance. Mol. Cell 4, 597–609 (1999). 10549291 10.1016/s1097-2765(00)80210-5

[b39] Wang H. & Peng D. Q. New insights into the mechanism of low high-density lipoprotein cholesterol in obesity. Lipids Health Dis. 10, 176, doi: 10.1186/1476-511X-10-176 (2011). 21988829 PMC3207906

[b40] Bonvecchio A. . Overweight and obesity trends in Mexican children 2 to 18 years of age from 1988 to 2006. Salud Publica Mex. 51 Suppl 4, S586–594 (2009). 20464234 10.1590/s0036-36342009001000013

[b41] Martinez-Marignac V. L. . Admixture in Mexico City: implications for admixture mapping of type 2 diabetes genetic risk factors. Hum. Genet. 120, 807–819, doi: 10.1007/s00439-006-0273-3 (2007). 17066296

[b42] Matthews D. R. . Homeostasis model assessment: insulin resistance and beta-cell function from fasting plasma glucose and insulin concentrations in man. Diabetologia 28, 412–419 (1985). 3899825 10.1007/BF00280883

[b43] Garcia Cuartero B. . [The HOMA and QUICKI indexes, and insulin and C-peptide levels in healthy children. Cut off points to identify metabolic syndrome in healthy children]. An. Pediatr. (Barc.) 66, 481–490 (2007). 10.1157/13102513 17517203

[b44] Kavey R. E. . American Heart Association guidelines for primary prevention of atherosclerotic cardiovascular disease beginning in childhood. Circulation 107, 1562–1566 (2003). 12654618 10.1161/01.cir.0000061521.15730.6e

[b45] Kalra S., Gandhi A., Kalra B. & Agrawal N. Management of dyslipidemia in children. Diabetol. Metab. Syndr. 1, 26, doi: 10.1186/1758-5996-1-26 (2009). 19995441 PMC2794840

