# Supplementary material for: Association between PPAR-γ2 Pro12Ala genotype and insulin resistance is modified by circulating lipids in Mexican children
Source: Sci Rep. 2016 Apr 14;6:24472. doi: 10.1038/srep24472 (PMC4830984; doi:10.1038/srep24472)
Supplement: Supplementary Information [file srep24472-s1.pdf]

Supplementary Information

Association between *PPAR-γ2* Pro12Ala genotype and insulin resistance is modified by circulating lipids in Mexican children

Carolina Stryjecki[1], Jesus Peralta-Romero[2], Akram Alyass[1], Roberto Karam-Araujo[3], Fernando Suarez[2], Jaime Gomez-Zamudio[2], Ana Burguete-Garcia[4], Miguel Cruz[2,*], David Meyre[1,5]

[1]Department of Clinical Epidemiology and Biostatistics, McMaster University, Hamilton, ON, Canada

[2]Medical Research Unit in Biochemistry, Hospital de Especialidades, Centro Médico Nacional Siglo XXI del Instituto Mexicano del Seguro Social, Mexico City, Mexico

[3]Health Promotion Division, Instituto Mexicano del Seguro Social. Mexico City, Mexico

[4]Centro de investigación sobre enfermedades infecciosas. Instituto Nacional de Salud Pública. Cuernavaca, Morelos, Mexico

[5]Department of Pathology and Molecular Medicine, McMaster University, Hamilton, ON, Canada

Supplementary Table S1: Power calculation for the main effect of *PPAR-γ2* rs1801282 on BMI.

MAF	Beta	Sample Size (unadjusted)[a]	Sample Size (adjusted)[b]	Interaction Sample Size (unadjusted)[a]	Interaction Sample Size (adjusted)[b]
0.14	0.50	2296	4495	118	230
	0.60	1596	3119	79	155
	0.70	1169	2290	56	109
	0.80	894	1751	41	80
	0.90	706	1382	31	60

Abbreviations: MAF, minor allele frequency

[a] power calculation unadjusted for multiple testing (2 sided p-value = 0.05, 80% power)

[b] power calculation adjusted for multiple testing (2 sided p-value = 2.08×10^{-3}, 80% power)

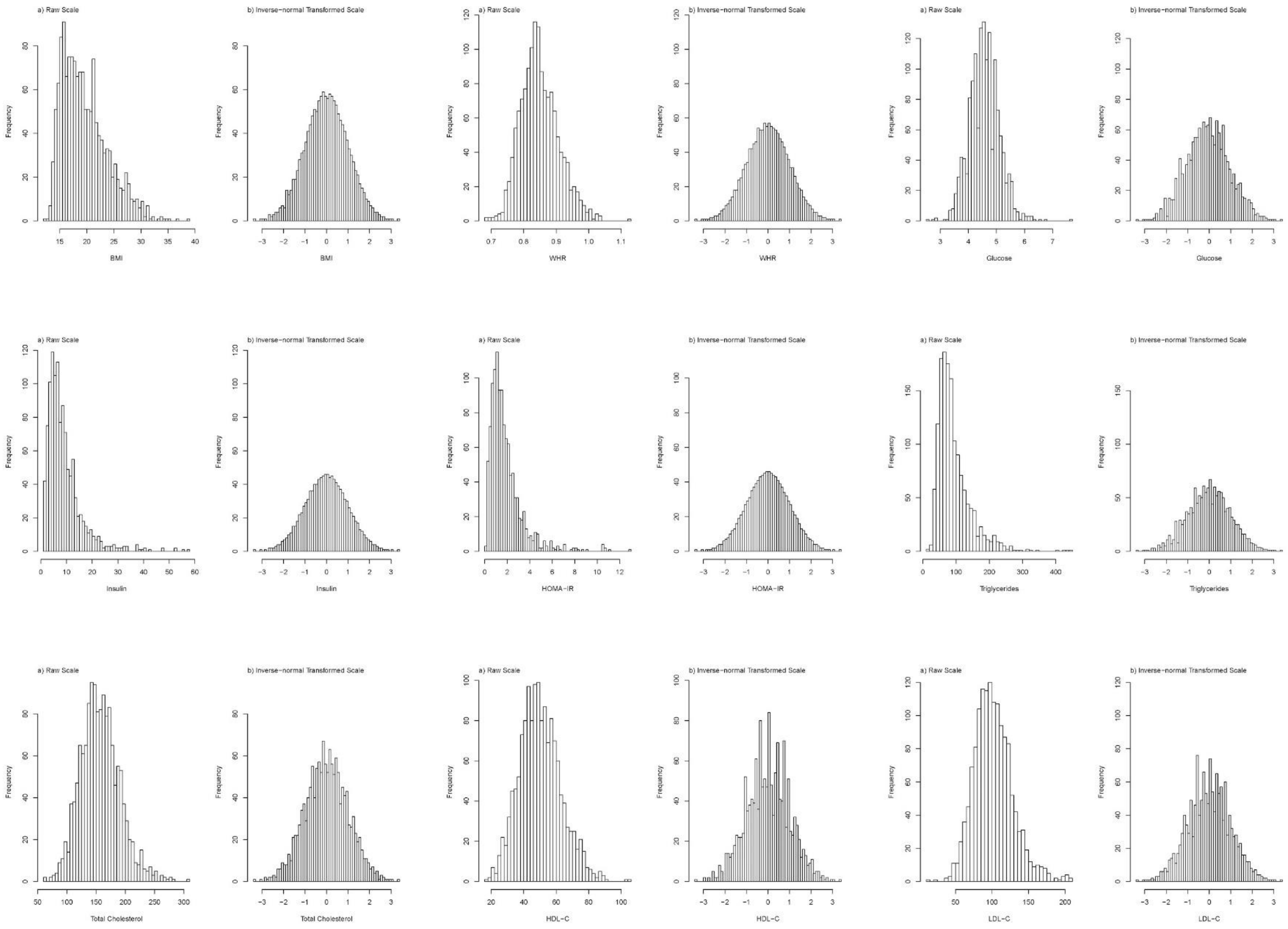

Supplementary Figure S1: Histograms illustrating raw distribution (panel A) and corrected distributions following inverse normal transformations (panel B) of variables of interest.
